# Retraction of: Validation of a popular consumer-grade cuffless blood pressure device for continuous 24 h monitoring

**DOI:** 10.1093/ehjdh/ztag002

**Published:** 2026-01-31

This is a retraction of: Bhavini J Bhatt, Haashim Mohammad Amir, Siana Jones, Alexandra Jamieson, Nishi Chaturvedi, Alun Hughes, Michele Orini, Validation of a popular consumer-grade cuffless blood pressure device for continuous 24 h monitoring, *European Heart Journal - Digital Health*, Volume 6, Issue 4, July 2025, Pages 704–712, https://doi.org/10.1093/ehjdh/ztaf044

In June 2025, the manufacturer of the wearable blood pressure monitor (W-BPM) notified the Journal of concerns regarding the data presented in the article. The Journal published an Expression of Concern^1^ while concerns about the authors’ interpretation of the W-BPM manual were investigated.

The Authors used only two accounts to collect data from 31 participants, whereas the device Instructions for Use state “Do not share your device with others as it may result in inaccurate blood pressure readings”.

The Editors are retracting this article because the interpretation of the manual and use of shared accounts renders the findings unreliable.

## Footnotes

1 Expression of Concern: Validation of a popular consumer-grade cuffless blood pressure device for continuous 24 h monitoring. European Heart Journal - Digital Health, https://doi.org/10.1093/ehjdh/ztaf134

